# Patterns and characteristics of ‘calls of despair’: a service evaluation using Yorkshire Ambulance Service data

**DOI:** 10.29045/14784726.2025.9.10.2.40

**Published:** 2025-09-01

**Authors:** Verity Bellamy, Holly Wilcock, Caitlin Wilson, Ruth Crabtree

**Affiliations:** Yorkshire Ambulance Service NHS Trust; Yorkshire Ambulance Service NHS Trust; Yorkshire Ambulance Service NHS Trust; University of Hertfordshire ORCID iD: https://orcid.org/0000-0002-9854-4289; Yorkshire Ambulance Service NHS Trust; Association of Ambulance Chief Executives

**Keywords:** death, emergency medical services, public health surveillance, substance-related disorders, suicide

## Abstract

**Introduction:**

‘Deaths of despair’ (DoD) – encompassing fatalities from suicide, drug overdoses and alcohol-related causes – represent a growing public health crisis. Socioeconomic vulnerability and healthcare disparities are well-documented drivers of DoD. While healthcare contacts preceding despair-related deaths have been studied extensively, the role of ambulance services is underexplored. This study aimed to address this gap by utilising ambulance service data to provide insights into ‘calls of despair’ received by a UK ambulance service over a 12-month period.

**Methods:**

This exploratory, retrospective study analysed data collected during 2023 by Yorkshire Ambulance Service (YAS), which serves urban and rural areas with varying levels of deprivation. Calls were included if they involved suicidal ideation and/or drug or alcohol misuse. Data were sourced from computer-aided dispatch and electronic patient records and were analysed to describe call characteristics, demographic profiles, geographical distribution, temporal trends and repeat caller patterns.

**Results:**

In 2023 YAS received 40,870 calls of despair. Nearly half of those calls originated from the most deprived quintile. Urban areas had more than double the rate of calls compared to rural areas. More than half (54%) of the calls involved drug and alcohol misuse, while 43% were related to suicidal ideation. Females were more likely to call for substance misuse (58%) than suicide (46%), and young females (<25 years) represented a disproportionate share of calls. Only 43% of calls resulted in hospital conveyance, suggesting ambulance services capture crises that are not reflected in hospital datasets. Repeat callers were common, with 119 individuals making more than 10 calls each.

**Conclusion:**

The findings highlight the utility of ambulance service data in understanding despair-related crises, particularly among socioeconomically disadvantaged and young populations. Ambulance data offers a valuable lens for public health monitoring, capturing acute needs often absent in traditional healthcare datasets. These insights emphasise the need for targeted interventions and cross-sectoral approaches to address the underlying drivers of despair.

## Introduction

‘Deaths of despair’ (DoD) is a term collectively used to describe fatalities due to suicide, drug overdoses and alcohol-related causes (Case & Deaton, 2017). These deaths disproportionally affect socioeconomically deprived communities and have become a growing public health crisis internationally (Beseran et al., 2022; Case & Deaton, 2022). In both the United Kingdom and the United States, research consistently shows that DoD is closely linked to socioeconomic vulnerability, unemployment and a lack of access to quality healthcare (Dobson et al., 2021; Thorndike et al., 2023; Walsh et al., 2021).

In England, between 2019 and 2021, 46,200 people died due to causes classified as DoD, averaging 42 lives lost each day (Camacho et al., 2023). Regional disparities in mortality rates were notable, with higher rates in the North of England. Socioeconomic factors, such as unemployment, urbanisation and a high proportion of White British populations, were strongly correlated with these deaths (Camacho et al., 2023). This pattern underscores the complex interplay of social determinants of health in driving mortality from despair.

Although alcohol, drug and suicide deaths have different aetiologies, they are interrelated in several critical ways. Alcohol and drug misuse are key risk factors for suicide, with intentional overdoses frequently involving a combination of drugs and alcohol (Camacho et al., 2023). Furthermore, individuals struggling with substance-use disorders often experience severe mental health issues, including depression and hopelessness, which heighten their risk of suicidal behaviour (Brådvik, 2018). This interplay of factors highlights the importance of examining despair-related deaths as a unified public health issue rather than isolated phenomena.

Efforts to understand and prevent DoD have increasingly focused on healthcare contact prior to these events. Studies have shown that help-seeking behaviour frequently occurs in those at risk of suicide and escalates in the weeks preceding death. For example, a Welsh study found that 73% of individuals who died by suicide had recent healthcare contacts, with 17% attending emergency departments in the months prior to death (John et al., 2020). Similar findings from France revealed that 61% of suicide decedents had recent healthcare contact, while 24% had been hospitalised (Laanani et al., 2020). These findings suggest a crucial window for intervention. However, most of these studies focus on hospital or primary care contacts and exclude ambulance calls – ‘calls of despair’ (Thorndike et al., 2023) – which may lead to an underestimation of prior help-seeking behaviour in despair-related crises.

Ambulance services often serve as the first point of contact for individuals in acute distress, providing a unique opportunity to study patterns of despair-related crises in real time. A report from Public Health Scotland (2024) on healthcare contacts prior to suicide found that unscheduled care – including ambulance calls – accounted for the highest number of contacts in the month before death. In the United States, studies by Dobson et al. (2021) and Thorndike et al. (2023) have successfully leveraged ambulance service data to track public health trends, including opioid overdoses and mental health crises, thereby demonstrating the value of such in understanding and addressing DoD. These studies underscore the potential of ambulance data to serve as a valuable early warning system, capturing critical moments of acute need that may otherwise be missed in hospital-based analyses.

In addition to studies on healthcare contacts, research in related fields has explored a variety of interventions aimed at preventing or mitigating deaths of despair. For instance, several countries have implemented harm-reduction strategies for substance misuse, such as supervised injection sites and increased access to naloxone, which has been shown to reduce overdose fatalities (Hawk et al., 2015; Sweileh, 2024). Additionally, community-based mental health initiatives, such as suicide prevention hotlines and outreach programmes, have demonstrated promise in reducing suicide rates (Mann et al., 2021). Moreover, policies aimed at improving social safety nets, such as unemployment benefits and universal healthcare access, have been linked to reductions in suicide and overdose deaths in some regions (Case & Deaton, 2020; Shand et al., 2022; Stack, 2021). These interventions, while not directly related to ambulance service data, highlight the multifaceted nature of efforts to prevent DoD and reinforce the importance of a holistic approach to tackling this public health crisis.

Despite the growing recognition of ambulance services as key responders in despair-related emergencies, there remains a significant research gap in the systematic study of ‘calls of despair’ in England. While hospital and primary care data have been extensively studied, ambulance service data remain an underutilised resource, limiting the ability of public health initiatives to fully understand the epidemiology of DoD and develop timely interventions. This exploratory study seeks to address this gap by utilising ambulance service data to provide novel insights into despair-related emergencies in England. The primary aim was to explore the characteristics and patterns of ‘calls of despair’ received by one UK ambulance service over a 12-month period. Specifically, the study sought to:

- Quantify the number of calls meeting the definition of ‘calls of despair’, including those related to suicidal ideation, drug and alcohol misuse or both.
- Describe the demographic profile (age, sex and ethnicity) of patients making such calls.
- Examine the outcomes of calls of despair, including patient disposition (e.g. transport to hospital), and the relationship between outcomes and patient demographics or call characteristics.
- Analyse the geographical distribution of calls, including urban versus rural areas, and areas of deprivation.
- Identify temporal trends, including patterns by day of the week and time of day.
- Assess the frequency of repeat calls and characterise patients making multiple calls.

## Methods

### Study design

This was an exploratory study using a retrospective review of routinely collected ambulance service data. The study aimed to identify and describe patterns in emergency calls related to ‘calls of despair’ over a 12-month period. The study size was not predetermined, as the objective was to capture the full range of relevant calls within the selected time frame. The 12-month period was selected pragmatically to include the most recent and complete data available at the time of analysis. This time frame allowed for seasonal variations and patterns in calls to be observed.

### Setting

Yorkshire Ambulance Service (YAS) NHS Trust covers a large and diverse region in Northern England, including urban and rural areas with varying levels of deprivation. Calls to YAS are categorised into three main outcomes: ‘hear and treat’ (resolved over the phone without dispatch), ‘see and treat’ (attended by ambulance but not conveyed to hospital) and ‘conveyed to hospital’.

### Inclusion criteria

Ambulance service data for Yorkshire and the Humber were reviewed for the period from January to December 2023. Calls were included if they met the predefined criteria for ‘calls of despair’, which encompassed calls related to suicidal ideation and drug and alcohol misuse. Only cases with linked electronic patient records (EPRs) were included in analyses where clinical impressions were required.

### Variables

The primary variables of interest included:

- **Call type:** Suicidal ideation, substance misuse or both.
- **Call outcome:** ‘Hear and treat’, ‘see and treat’ or ‘conveyed to hospital’.
- **Demographics:** Age, sex and geographical location of callers.
- **Temporal factors:** Day of the week and time of day of the call.
- **Deprivation level:** Index of Multiple Deprivation (IMD) 2015 quintile for the caller’s location.
- **Geographic area:** Local authority and lower super output area (LSOA) of the caller.

### Data sources

Data were obtained from two main sources: computer-aided dispatch (CAD) data and EPR data.

CAD data provided information on all emergency calls, including call type, outcome, timestamps and free-text fields. As CAD captures all ambulance calls made to the ambulance service, including those resolved without ambulance dispatch (‘hear and treat’), it was the primary data source for defining ‘calls of despair’.

EPR data were linked where available (n = 26,588, 65%) for attended calls, providing additional clinical details recorded by ambulance crews. EPR records included the main working impression coded using the ambulance dataset coding system, which categorises clinical impressions into hierarchical groups, from the broadest category (‘Group 1’) to the most specific diagnosis (‘Description’).

### Data extraction

A detailed coding framework was developed to classify calls into the categories of interest (Supplementary 1). Suicidal ideation calls were identified through specific dispatch codes that explicitly referenced that the call was a suicide attempt (e.g. ‘Falls – serious haemorrhage jumper (suicide attempt)’) or dispatch codes that specify self-inflicted intent (e.g. Self-inflicted knife/stab wound (intentional)’). Keyword searches (e.g. suicid*) were applied to the free-text fields to capture additional cases. The exception to this were codes that related to intentional or self-inflicted overdose/poisoning, as preliminary analysis of the free-text field suggested that ‘self-inflicted’ and ‘intentional’ in this context frequently indicated that the patient had intended to take the substance but not necessarily to overdose on it. These calls were included in the overall ‘calls of despair’ under substance misuse calls.

Substance misuse calls were identified using dispatch codes and chief complaints related to overdose, poisoning and alcohol/drug misuse.

Structured queries using SQL were used to automate data extraction based on the criteria outlined. Age fields were cleaned and data were linked to LSOA (LSOA11) and IMD2015 via the postcode field recorded at time of ambulance dispatch. Manual validation and review of free text was performed on a subset of records to ensure accuracy. Data were then extracted into Microsoft Excel and summarised using pivot tables, as well as rates and charts created in Excel.

### Data analysis

Descriptive analysis was used to summarise the frequency and characteristics of calls, including patient demographics, time and day of calls and geographical distribution. The analysis also examined the proportion of calls that resulted in ‘hear and treat’, ‘see and treat’ or hospital conveyance. Rates of calls were calculated per 1000 population using publicly available population estimates. For patients with repeat calls, NHS numbers were used to identify distinct individuals. Only calls with valid NHS numbers were included in the repeat call analysis. The analysis was undertaken in Excel and SQL.

Geographical analysis was conducted by mapping calls to local authorities and LSOAs using postcode data. Rates of calls by rural/urban classification and IMD quintile were calculated to assess potential disparities in call distribution. LSOAs were categorised using the Office for National Statistics (ONS, 2021) rural urban classification. National IMD quintiles were used to align with NHS England’s CORE20PLUS5 framework, which focuses on the most deprived 20% of the national population (NHS England, 2021). Using national rather than local IMD quintiles allows for consistency with national policy and benchmarking. To account for population differences, call rates rather than raw counts were used in the analysis.

## Results

**Figure 1:**
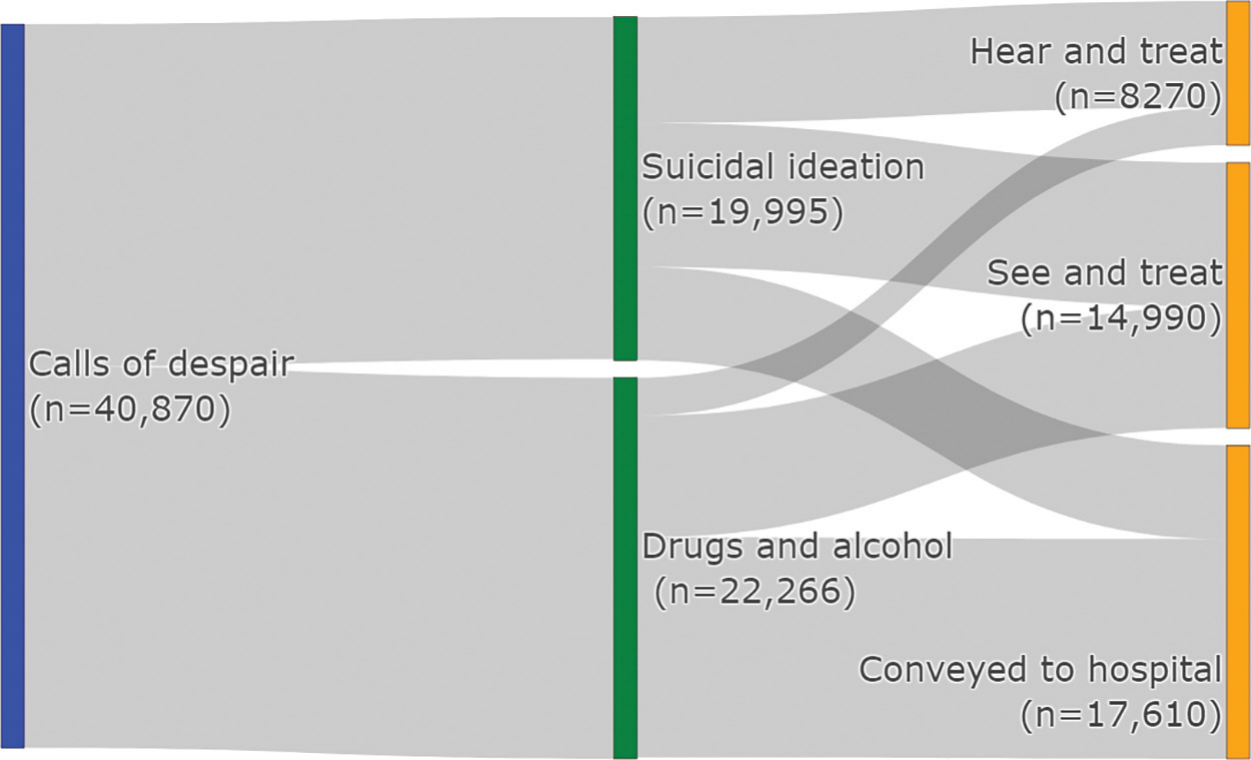
Figure 1. Sankey diagram indicating calls of despair outcomes.

In 2023 there were 40,870 calls to YAS that met our definition of calls of despair (Figure 1). A fifth of these (n = 8270) were ‘hear and treat’, 37% (n = 14,990) were ‘see and treat’ and 43% (n = 17,610) were ‘conveyed to hospital’. This means 57% of the patients in despair (n = 23,260) were not conveyed to hospital and their episode of care may not be known to other parts of the system.

Of all calls, 54% (n = 22,266) met the definition for calls relating to drugs and alcohol, and 49% (n = 19,995) met the definition for suicidal ideation, with 3% (n = 1391) of calls meeting both definitions. Patients who met the drug/alcohol definition were more likely to be conveyed to hospital (58%, n = 12,845), compared to 27% (n = 5462) of those with suicidal ideation. Those who were suicidal were more likely to be a ‘see and treat’ (42%) or ‘hear and treat’ (31%), meaning there were around 14,500 patients in 2023 who were suicidal and may have only been seen by the ambulance service. In comparison, a third (32%) of drug/alcohol calls were a ‘see and treat’ and 10% were a ‘hear and treat’.

### Age and sex

**Figure 2:**
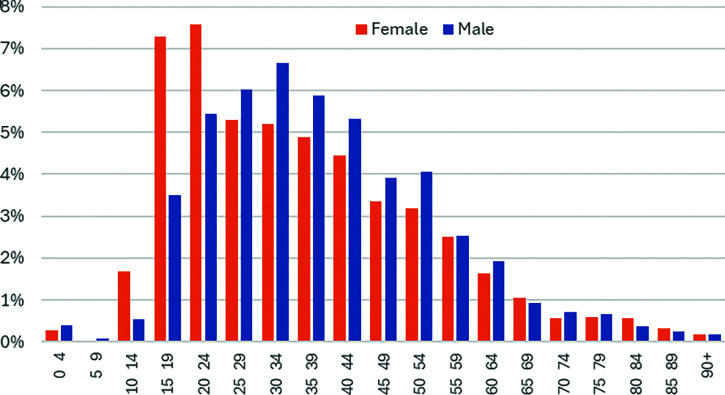
Figure 2. Calls of despair by age and sex.

Among those under 30, 58% of calls were for drugs and alcohol, compared to 45% for suicidal ideation (Figure 2). As patients aged, the proportion of calls related to drugs and alcohol declined, with 55% of calls in the 30–44 age range being for drugs and alcohol and 49% for suicidal ideation. However, the proportion of calls for suicidal ideation increased with age, reaching 52% of calls for those aged 45 and over, compared to 50% for drugs and alcohol. This suggests a shift in the nature of the calls as individuals age, with a relative increase in suicidal ideation calls in older age groups.

Overall, there was a very similar proportion of calls of despair for males (50%, 20,012) and females (50%, 20,328). Male calls had a similar proportion meeting the drug/alcohol and suicidal ideation definitions, with 52% meeting each definition (3% meeting both). Female calls were more likely to be for drugs/alcohol (58%) than suicidal ideation (46%). Females were more likely to be conveyed (45%) than males (41%).

Calls were predominantly from younger callers, with more than three quarters (78%) of all callers under 50 and half (50%) under 35. There is an apparent spike in younger females, with 17% of all calls and a third of all female calls (33%) from those aged under 25. Females who called were generally younger than males, with a female mean age 35 (median 32) and male mean age 38 (median 35).

The mean age was younger for calls relating to drugs and alcohol (34 for females, 37 for males) compared to calls relating to suicidal ideation (36 for females, 39 for males).

### Rural versus urban

**Figure 3:**
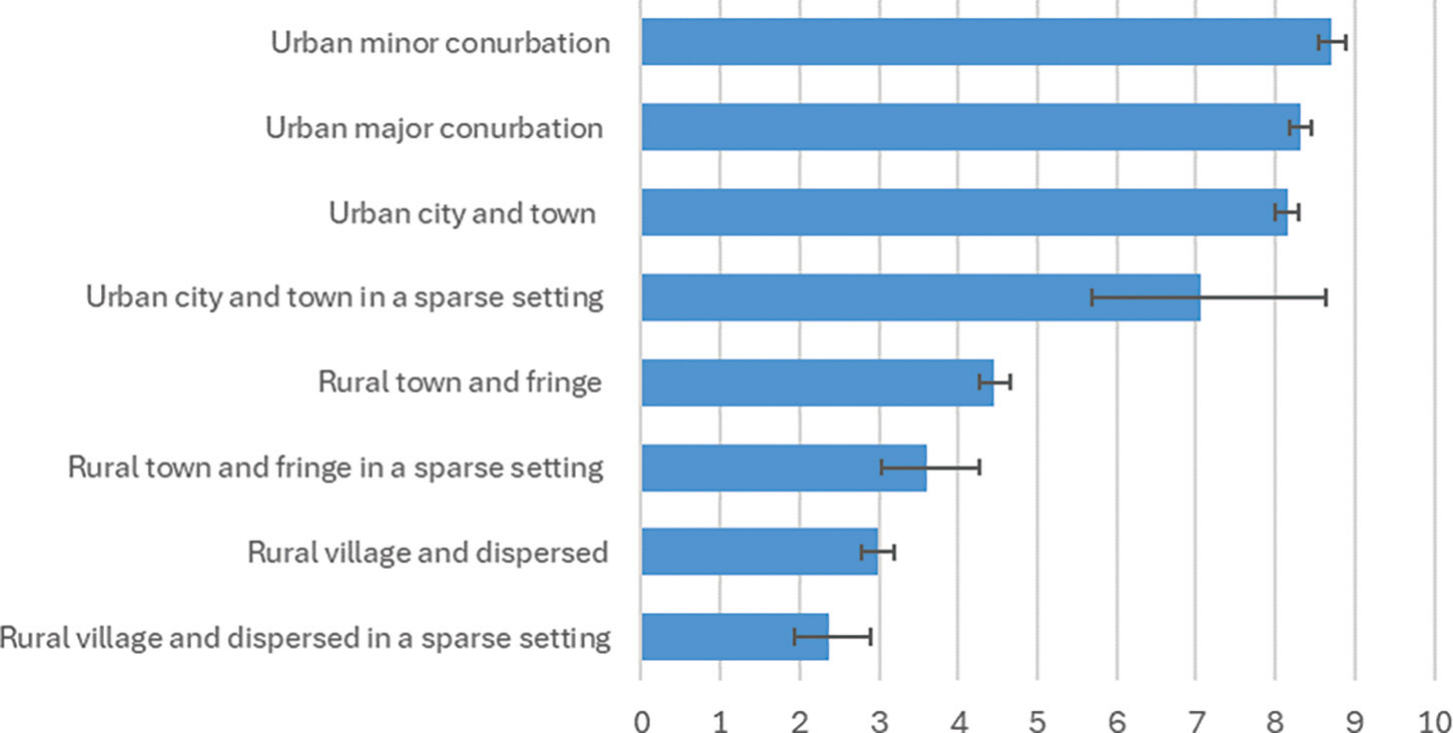
Figure 3. Ambulance calls of despair by rural urban classification.

Most of the population of Yorkshire and the Humber live in an urban area (84%), compared to 16% in a rural area. However, urban areas were still over-represented in calls of despair, with 89% (n = 36,559) from an urban area compared to 8% from a rural area (n = 3133); the rate of calls in urban areas (8.4 calls per 1000 population) was more than twice that in rural areas (3.8 calls per 1000 population) (Figure 3).

The ONS also provides a more detailed categorisation of urban/rural groups, which shows even greater variation in the rate of calls of despair. The rate of calls in the ‘urban minor conurbation’ category is significantly higher than in the ‘urban major conurbation’ or ‘urban city and town’ categories (see Figure 3). It is also more than three and half times that in the ‘rural village and dispersed in a sparse setting’.

### By IMD

**Figure 4:**
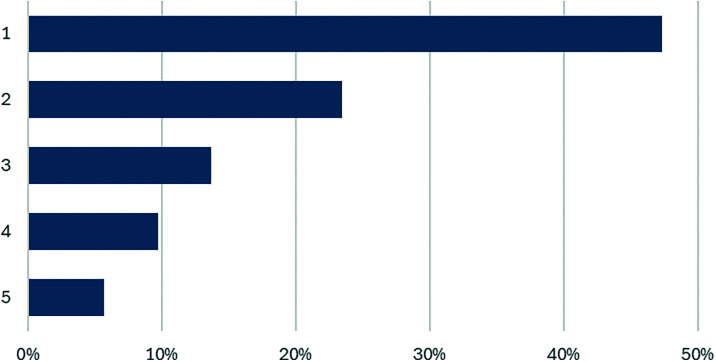
Figure 4. Calls of despair by quintile of deprivation.

In Yorkshire and the Humber, around 29% of people live in the most deprived quintile nationally and 15% in the least deprived. Almost half of all calls of despair were from the most deprived quintile (47%) and only 6% from the least deprived quintile, with the rate of calls of despair in the most deprived quintile more than four times that in the least deprived (Figure 4).

### Temporal trends

Table 1 illustrates that calls of despair were more likely to occur over the weekend, with the highest proportions on Friday (14.8% of calls) and Saturday (14.9% of calls). This trend was primarily driven by drug- and alcohol-related calls, which showed a noticeable increase over the weekend, with 14.9% of calls on Friday, 15.8% on Saturday and 15.4% on Sunday, compared to weekdays, where an equal distribution would result in approximately 14.3% of calls each day. There was no noticeable difference between days of the week for calls meeting our definition of suicidal ideation.

**Table 1.** Calls of despair by day and time.

	Time of day	Monday	Tuesday	Wednesday	Thursday	Friday	Saturday	Sunday
All calls	0–2	669	650	670	663	708	924	1038
	3–5	326	294	293	351	385	548	613
	6–8	354	286	300	305	296	332	339
	9–11	590	650	622	663	687	530	527
	12–14	784	822	828	812	797	673	710
	15–17	957	918	985	970	962	827	785
	18–20	981	1008	1035	1008	1061	1043	967
	21–23	966	966	968	1030	1153	1211	1030
Suicidal ideation	0–2	327	312	298	330	347	384	414
	3–5	166	159	138	180	189	253	265
	6–8	177	139	125	149	150	169	161
	9–11	301	340	316	338	334	240	241
	12–14	391	434	436	392	390	312	353
	15–17	509	497	502	494	506	400	359
	18–20	471	509	548	523	508	503	498
	21–23	489	511	478	519	521	515	485
Drugs/alcohol	0–2	368	361	396	354	394	564	652
	3–5	170	145	163	186	211	312	360
	6–8	184	153	180	162	153	177	185
	9–11	308	330	330	339	371	304	302
	12–14	421	415	419	440	428	390	382
	15–17	482	455	514	516	490	447	451
	18–20	546	526	533	528	596	581	508
	21–23	506	491	527	560	677	738	585

Calls of despair were predominantly made between 18:00 and 23:00, with over a third (35.3%) of calls between these times. Almost two thirds of calls (64%) occurred between 15:00 and 02:00. These trends were similar across all types of calls.

### Repeat calls

Of the 40,870 calls of despair overall, 53% (n = 21,656) had a valid NHS number recorded within the data and, of these, 14,822 were distinct NHS numbers and therefore different individuals. Over 12,000 individuals had only one call. However, this means that 2662 patients had more than one call of despair recorded. These 2662 patients accounted for 9500 calls.

Of patients with more than one call, 58% had two calls recorded and 17% had three calls recorded. There were another 12% (n = 310) who had between five and nine calls recorded meeting the definition of calls of despair, 4% (n = 119) with more than 10 calls and 1% (n = 34) with more than 20 calls. The 119 patients with more than 10 calls accounted for a total of almost 2000 calls (n = 1983) and the 34 patients with more than 20 calls accounted for 1067 calls in total.

**Figure 5:**
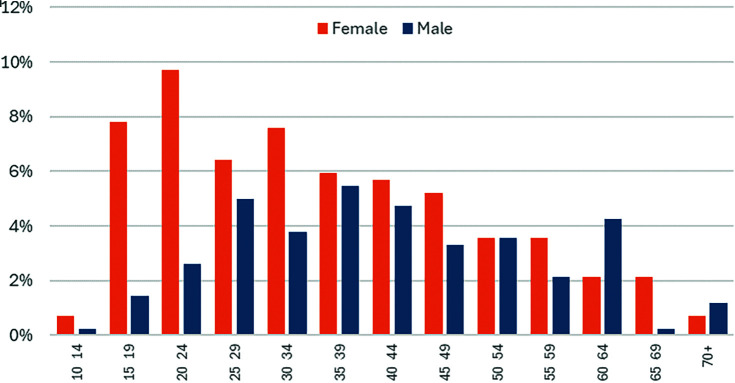
Figure 5. Patients with five or more calls of despair by age and sex.

Figure 5 highlights that patients with five or more calls were more often females (61%, n = 258) than males (38%, n = 160). A quarter of all the repeat calls were women under the age of 30.

## Discussion

This exploratory study highlights the utility of ambulance service data in understanding despair-related emergencies in England, offering novel insights into the scale, demographics, geographical distribution and temporal trends of such calls. Over 40,000 calls of despair were recorded in 2023 by YAS, with drug- and alcohol-related calls comprising the majority (54%). These findings align with the study’s primary aim to quantify, describe and analyse the characteristics and patterns of despair-related emergencies.

The study revealed that fewer than half (43%) of patients experiencing calls of despair were conveyed to hospital, with an even lower proportion (27%) among suicidal patients. Similar to findings from Public Health Scotland (2024), the low proportion of hospital conveyance among these patients suggests that many instances of acute distress do not escalate to acute healthcare settings. This underscores the critical role ambulance services can play in identifying and supporting individuals at risk, filling a gap in the continuum of care that is often missed by hospital-based studies.

Demographic analysis showed that despair-related emergencies affect both males and females in near-equal proportions; however, females were more likely to call for drug- and alcohol-related issues (58%) than for suicide (46%). Young adults were disproportionately represented, with half of all calls from individuals under 35 and a notable spike among females under 25. This demographic focus aligns with findings by Camacho et al. (2024), which highlighted age-related variations in DoD, particularly the prominence of suicide among younger age groups.

In some circumstances, it is possible that calls for drugs and alcohol to the ambulance service may reflect more recreational use than the deaths reported in the Camacho et al. (2024) study. However, the necessity for a call to an ambulance service suggests that this use is, nonetheless, at a harmful level.

The study identified links between calls of despair and socioeconomic deprivation. Nearly half (47%) of all calls originated from the most deprived quintile, with rates more than four times higher than in the least deprived quintile. Calls were also more prevalent in urban areas, with rates in urban, minor conurbations exceeding those in rural areas by more than twofold. These findings mirror the geographical and socioeconomic patterns reported by Camacho et al. (2024), underscoring the intersection between deprivation and despair-related emergencies. The predominance of urban areas as hotspots for calls mirrors patterns observed in the United States, where urbanisation is closely tied to the prevalence of opioid overdoses and mental health crises (Dobson et al., 2021). The UK Chief Medical Officer’s annual report (Whitty, 2024) further highlights the importance of tailoring healthcare service planning and delivery to the needs of young adults in cities, whose mental health issues – often emerging in early adulthood – disproportionately strain already overstretched urban mental health services.

Although not all individuals who make suicide attempts die by suicide, this study showed that there are a number of patients who make multiple ‘calls of despair’. A particularly concerning finding was the frequency of repeat calls: over 2500 patients made more than one call in 2023, with 119 patients making more than 10 calls and 34 patients making more than 20 calls. This aligns with existing literature on frequent users of emergency medical services, who often experience complex health and social challenges (Gehrt et al., 2025; Snooks et al., 2019). Targeted interventions for these individuals could reduce service demand while addressing underlying causes of despair (Aslam et al., 2022; Jones et al., 2025).

### Limitations

This study has several limitations that should be considered.

Ethnicity data were incomplete, with over half of the EPRs missing this information. Only 44% of patients were recorded as White British, and fewer than 1% as other ethnicities, limiting the ability to assess ethnic disparities.

Geographical analyses used the call location rather than the patient’s home address. While this identifies where emergencies occur, it may not accurately represent the caller’s socioeconomic or geographical background, particularly for calls from transient or central urban locations.

IMD2015 rather than the more current IMD2019 was used due to only the former being available in the data warehouse. While the chosen national IMD quintiles align with NHS England’s CORE20PLUS5 framework, this approach does not account for local deprivation distributions, which may skew regional comparisons.

The study relied on retrospective data from routine ambulance operations. Inconsistent coding in free-text fields and dispatch codes may have led to misclassification. Additionally, repeat call analysis was limited by incomplete NHS number recording, with only 53% of calls containing valid identifiers, likely underestimating repeat callers.

Routine data collected by the ambulance have limitations in defining this population accurately and, particularly when dealing with drugs and alcohol, the intent of the patient is difficult to ascertain.

The analysis period was pragmatically chosen, limiting insights into long-term trends. Findings may not generalise beyond Yorkshire and the Humber. The study also lacked follow-up data on outcomes such as hospital admissions, referrals or deaths, which restricted the ability to assess the broader impact of calls.

### Recommendations for research and practice

This study highlights key opportunities to improve understanding and intervention for despair-related emergencies. Integrating ambulance service data with health and social care records is essential to capture patient trajectories, including follow-up care, referrals and long-term outcomes. Addressing disparities in deprived areas and urban centres is also crucial. Small-area mapping can guide targeted community mental health and substance misuse initiatives, reducing demand on emergency services.

There is a need to rethink emergency responses for individuals in crisis, particularly those experiencing acute despair. The perceived immediacy of an ambulance often makes it the first choice, even when alternative crisis care may be more appropriate. This study highlights the mismatch between emergency medical responses and the social and mental health needs of these individuals. Integrating crisis teams, community-based support and improved triage at the point of call could provide more tailored care while reducing pressure on ambulance services.

Given the disproportionate impact on young people, especially females under 25, youth-focused mental health and outreach programmes through schools and universities should be prioritised. For frequent callers, multidisciplinary care plans involving mental health professionals, social workers and addiction specialists can address unmet needs and reduce the strain on emergency services.

Seasonal and temporal variations in calls of despair warrant further investigation. While this study focused on 2023, supplemental data suggest a drop in calls during winter 2022, followed by a sharp increase in the New Year. This may reflect seasonal factors like weather, holidays or financial pressures. Overall, calls of despair increased by 12% in 2023 (n = 40,870) compared to 2022 (n = 36,363). Preliminary 2024 data (up to November) already show a higher total (n = 41,433) than in 2022 or 2023. These trends highlight the need for ongoing monitoring to better understand seasonal and year-on-year variations and inform resource planning.

Finally, improvements in data collection are critical. Addressing gaps in ethnicity recording, ensuring consistent coding and increasing NHS number capture will improve the reliability of future research and support equitable interventions, particularly for under-represented groups.

## Conclusion

This study demonstrates the critical role of ambulance services in responding to despair-related emergencies and capturing data on vulnerable populations who may be invisible to other healthcare systems. The findings highlight patterns between calls of despair and deprivation, urbanicity and younger age groups. Despite limitations, these results offer a valuable foundation for informing targeted interventions and guiding future research. By leveraging ambulance data alongside linked datasets, healthcare systems can develop more effective strategies to reduce DoD and improve support for individuals in crisis.

## Author contributions

RC and VB designed the study. VB and HW completed data collection and analysis. RC and CW supported with interpretation of the data. VB drafted the report underlying this manuscript. CW drafted the first version of the manuscript. All authors contributed substantially to its revisions and approved the final manuscript. VB acts as the guarantor for this article.

## Conflict of interest

CW is an associate editor of the BPJ.

## Ethics

Not required.

## Funding

None.
